# Triplet Excitons in Carbon Nitride Materials: From Melem Monomers to Extended Polymers

**DOI:** 10.1021/acs.jpclett.5c02668

**Published:** 2025-10-12

**Authors:** Arianna Actis, Niccolò Ulivieri, Lorenzo Poggini, Mario Chiesa, Enrico Salvadori

**Affiliations:** ∇ Dipartimento di Chimica, Università di Torino, Via P. Giuria 7, 10125 Torino (TO), Italy; ‡ CNR, ISTITUTO DI CHIMICA DEI COMPOSTI ORGANOMETALLICI, Via Madonna del Piano 10, 50019 Sesto Fiorentino (FI), Italy; § Department of Chemistry ‘Ugo Schiff’ DICUS, and INSTM Research Unit, University of Florence, Via Della Lastruccia 3-13, 50019 Sesto Fiorentino, Italy

## Abstract

Owing to their earth-abundant constituents and tunable electronic structure, Carbon Nitride (CN) materials have emerged as a versatile class of photocatalytic systems. Their photocatalytic activity is linked to a complex excited-state landscape, where the role of triplet excitons remains under debate. In this work, we use time-resolved electron paramagnetic resonance (TR-EPR) spectroscopy to directly probe the spatial extent of triplet excitons as a function of the degree of thermal polymerization. We find that fully polymerized CN frameworks sustain strongly delocalized triplet excitons, in contrast to the localized states observed in incompletely condensed structures containing molecular or oligomeric units. These results establish a clear connection between network polymerization and exciton delocalization, providing mechanistic insight into the photophysical processes that govern charge separation and reactivity in CN-based photocatalysts.

## Graphical abstract

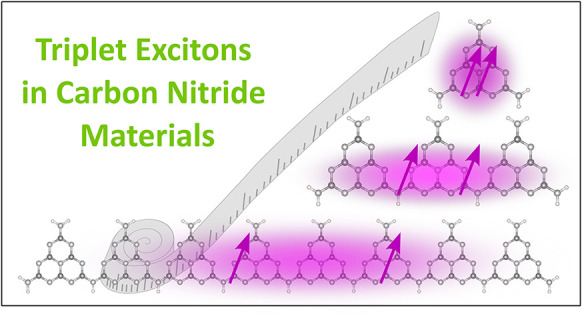

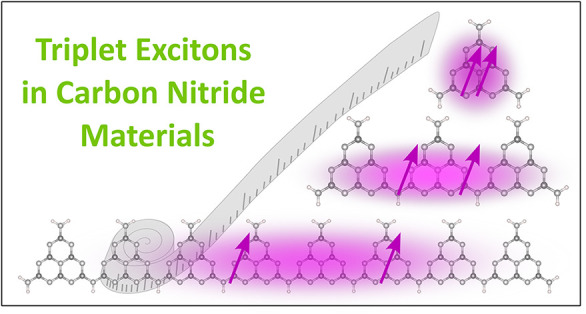

Photochemical transformations are often promoted by photocatalysts, which can be either homogeneous or heterogeneous. While homogeneous photocatalysis provides a high density of redox active sites per volume unit, the properties of heterogeneous photocatalysts, typically semiconductors, can be tailored by adjusting their morphology. The photophysics and photochemistry of homogeneous catalysts, represented, for instance, by molecular entities such as organic dyes or transition metal complexes, are dominated by localized excited states, among which triplet excited states (*S* = 1) are often involved.^1,2^ On the other hand, while discussing the photophysics and photochemistry of solid-state inorganic semiconductors, it is generally not appropriate to invoke the involvement of triplet states, as these excited states are typically delocalized across the whole crystal lattice. A class of materials that sits at the intersection of these two extremes is composed of π-conjugated systems. They bridge homogeneous and heterogeneous catalysts by combining the structural tunability and defined active sites typical of molecular catalysts with the solid-state robustness and recyclability of traditional heterogeneous systems.

Carbon nitride (CN) is an example of a π-conjugated polymeric semiconductor that has gained considerable interest in the past decades for its ability to promote a large variety of photoredox processes.^3^ CN has an ideal formula, C_3_N_4_, and is constituted by heptazine or triazine units cross-linked via nitrogen bridges and stacked in a graphitic fashion.^4,5^ In the literature, CN is often called “graphitic carbon nitride”, or g-C_3_N_4_.^4^ However, the stoichiometry of the actual materials differs from the ratio expected for true C_3_N_4_ and invariably contains a substantial amount of hydrogen (≈2 wt %). The structure of CN is therefore more consistent with Liebig’s melon - ideal structural formula C_6_N_9_H_3_ ^6^ or more correctly C_6_N_7_(NH)­(NH_2_) - which corresponds to 1D polymeric chains held together by hydrogen bonds.^7^ CN is commonly synthesized through a thermal condensation process that is not homogeneous and may vary throughout the sample. As a consequence, a single batch of CN material usually displays variable degrees of polymerization, whereby short oligomers coexist with extended polymerized regions.^7,8^ Hence, all spectroscopic investigations are complicated by the poor solubility, the presence of structural defects, and the undefined chain length. Lotsch and co-workers^9^ conducted a systematic study on the identity and structure of small condensates, which were identified as oligomers of heptazine, the monomeric unit constituting CN. When isolated and tested singularly, these species showed improved activity in sacrificial hydrogen production as compared to polymeric CN. Schlenker et al. reported that when such structures are present in a mixture with polymeric CN, they enhanced the extraction of charge carriers from bulk CN and facilitated the transport toward reactants.^10^ From these studies it appears that components with different degrees of polymerization in CN are able to act cooperatively. Although the chemical and redox properties of heptazine oligomers have been investigated,^9–11^ much less attention has been paid to their excited states, in particular to the triplet excitons. Triplet excitons play a key role as precursors of charge carriers. In fact, due to their spin forbidden radiative recombination, triplet excitons have a longer lifetime compared to singlet excitons.^12^ The longer lifetime enables a higher diffusion length within the material, with higher probability to reach the surface, as demonstrated by their ability to balance the rate asynchrony between reduction and oxidation events in a photocatalytic process.^13–15^ Furthermore, heptazine derivatives and CN materials are characterized by an inverted singlet–triplet energy gap.^16–23^ This property implies (i) that in such materials the generation of excitons in a triplet state is not a downhill process, which reduces efficiency losses; and (ii) that there exists a complex interplay between singlet and triplet excitons. Recent works on a g-C_3_N_4_-quantum dot heterojunction have assessed the critical role of the excited-state to enhance the efficiency of photocatalytic activity.^24,25^

Here, the properties of triplet excitons in CN materials are investigated as a function of the degree of polymerization using time-resolved EPR (TR-EPR) spectroscopy. We find that variations in the extent of polymerization are reflected in distinct triplet exciton characteristics. Specifically, the spatial extent of the excitons — estimated from the zero-field splitting (ZFS) parameter *D* — correlates with their degree of delocalization, which reflects the underlying macromolecular size, as independently inferred from photoluminescence measurements.

Three samples were considered, each characterized by a different degree of polymerization. The CN monomer (NH_2_-terminated heptazine or 2,5,8-triamino-tristriazine) was compared with a sample constituted by short oligomers and with polymeric CN. For consistency with the literature, the samples are hereafter labeled as *melem*, *oligomers* and *CN*, respectively. The synthesis was performed according to a bottom-up approach inspired by the work of Lotsch et al.,^9^ whereby different degrees of polymerization were achieved through a thermal condensation process carried out at different temperatures and for different reaction times (see SI Materials and Methods).

To confirm that the synthesis conditions yielded the expected materials, we collected diffuse reflectance UV–vis (DR-UV–vis), solid-state photoluminescence, attenuated total reflection IR (ATR-IR) spectra, and X-ray diffraction (XRD) patterns (Figure 1a–d and Figures S1 and S2). The spectral features are consistent with literature reports on analogous materials. The DR-UV–vis (Figure 1a) spectra show the clear progression from the well-defined absorption band of a molecular species (melem) to the characteristic spectrum of a semiconductor (CN). The blueshift of the onset of the absorption band is indicative of the progressive reduction of the dimension of the structure. The photoluminescence spectra (Figure 1b) show emission maxima at 405, 445, and 550 nm for melem, oligomer, and CN, respectively. Time-correlated single-photon-counting traces are reported in Figure S3. For all materials, the emission maximum quoted corresponds to the maximum of the component not altered by changing the excitation wavelength (Figure S1). In the ATR-IR spectrum of melem, the band attributable to the bending mode of secondary amine -NH (ca. 1230 cm^–1^) is absent (shaded area in Figure 1c), suggesting a predominance of monomeric structures in this sample.

**Figure 1:**
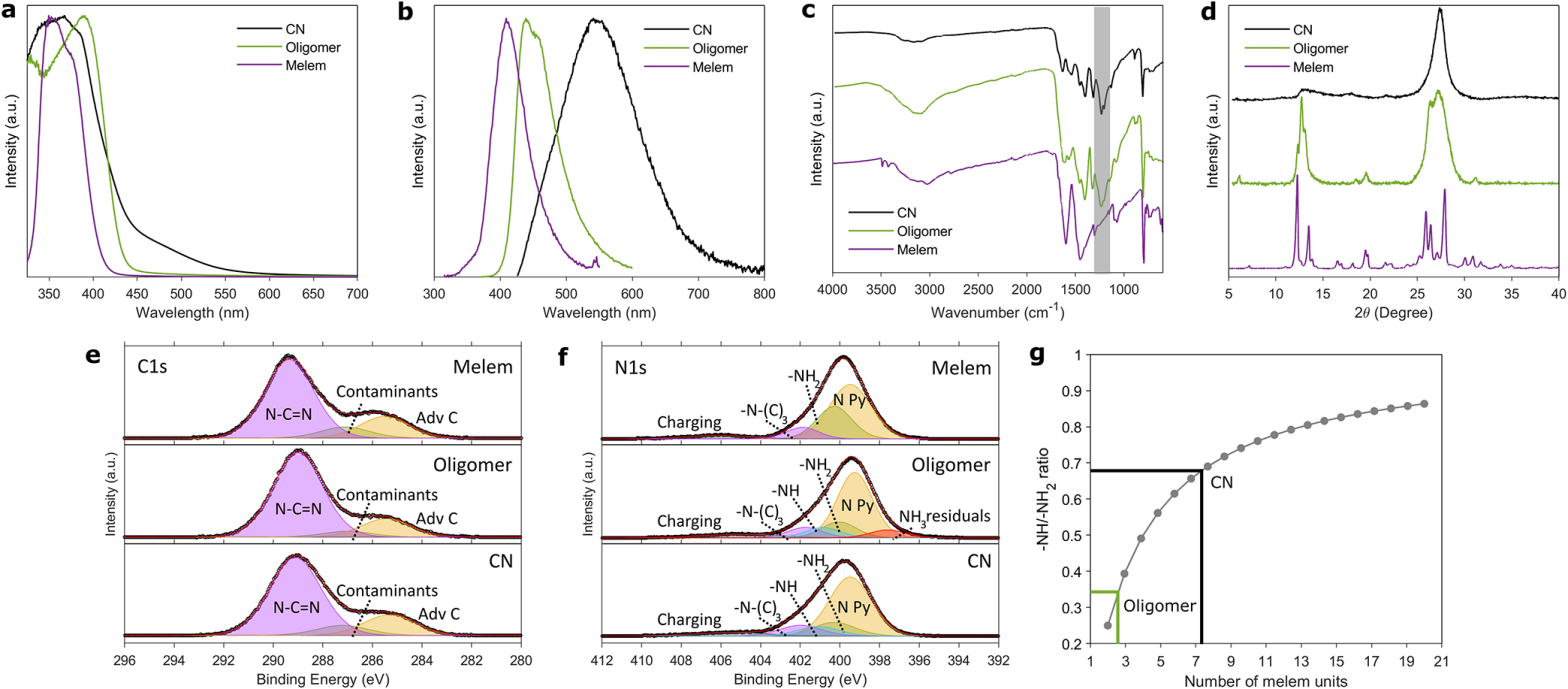
**a** – Normalized UV–vis diffuse reflectance spectra; **b** – Normalized photoluminescence spectra with λ_exc_ = 350 nm; **c** – ATR-IR spectra; **d** – Powder X-ray diffractograms for melem, oligomer and CN recorded at room temperature. **e** – XPS spectra of C1*s* and **f** – N1*s* of the CN materials as a function of polymerization. **g** – Theoretical dependence of the -NH/-NH_2_ ratio for a linear polymer as a function of the number of melem units. The green and black lines show the experimental ratios derived from XPS and the corresponding number of units.

However, comparison of the XRD patterns shows that the melem and oligomer samples contain a fraction of fully polymerized CN (Figure 1d and Figures S1 and S2). This is consistent with the thermal condensation process employed, which is not homogeneous and varies throughout the sample, making it difficult to fully control the effective polymerization.^4,26^ Therefore, to properly quantify the degree of polymerization induced by the thermal treatment, the samples were analyzed through X-ray photoelectron spectroscopy (XPS). An overview of the XPS spectral features corresponding to the C1*s* and N1*s* regions is presented in Figure 1e-f, and a comprehensive description of the semiquantitative analysis of the elements is provided in Table 1 and Table S2.

**Table 1:** Elemental Composition of Carbon Nitride Materials As Derived from XPS^a^

***Sample***	***C (%)***	***N (%)***	***C***_***tot***_ ***/N***_***tot***_	***C***_***C–N*** = ***C***_ ***/N***	***C/N***_***theor***_
***melem***	46.18	53.82	0.86	0.60	0.60
***oligomer***	44.76	55.24	0.81	0.65	0.63^a^
***CN***	46.69	53.31	0.88	0.69	0.66^b^

a The *C*_tot_/*N*_tot_ ratio considers all the C and N species, whereas the C _C–N=C_/N ratio takes into account only the carbon and nitrogen from the heptazine structure. The theoretical C/N ratio is reported in the last column for reference.

b Value calculated assuming an oligomer formed by 3 units.

c Value calculated assuming a 1D polymer.

From a structural standpoint, the C1*s* region provides limited insight to evaluate the progression from monomer to polymer due to the similarities across the samples, as shown in Figure 1e. For this reason, a detailed analysis of the C1*s* region is reported in the Supporting Information (section 3). In contrast, the analysis of the N1*s* peaks (Figure 1f) offers more meaningful differentiation, particularly through the emergence of the component corresponding to secondary amine groups (-NH, at ca. 401.14 eV), which is absent in the melem monomer and indicative of successful polymerization.

The actual degree of polymerization can be estimated via XPS by monitoring the presence and relative intensities of amino functionalities within the structure. By comparing the ratios between the areas of the secondary (-NH) and primary amine peaks (-NH_2_), it is possible to estimate the number of melem units present in the polymerized samples. A number of studies demonstrated the feasibility of this approach under the assumption of a linear polymer chain.^9,26^ For example, the molecular system resulting from the combination of two melem units (dimer) should contain four -NH_2_ groups and one bridging -NH group connecting the two cores (-NH/-NH_2_ = 0.25). Similarly, a trimer would be expected to contain five -NH_2_ groups and two primary -NH groups (-NH/-NH_2_ = 0.4). As the number of repeating units in the polymer increases, the NH/NH_2_ ratio should correspondingly increase, as shown in Figure 1g according to the relation -NH/-NH_2_ = n – 1/n + 2, where n is the number of heptazine units in the chain. Analysis of the XPS data provides for the oligomer a -NH/-NH_2_ ratio of 0.33, a value that falls between the theoretical ratios calculated for two- and three-unit systems. On the basis of the XPS data and the photoluminescence spectra reported in the literature for well-defined heptazine oligomers obtained through wet synthesis,^26^ we conclude that the oligomer is constituted on average by three melem units. In contrast, the CN sample exhibits a ratio of 0.69, which corresponds to an average length of eight units. Considering the edge-to-edge dimension of the heptazine (∼0.68 nm), for CN this amounts to a linear dimension of 5.4 nm, in excellent agreement with the value of 5.5 nm obtained for the crystallite size from the XRD peaks (Figure 1d) using the Scherrer equation (see Supporting Information section 1.2).

The presence and magnetic properties of triplet excitons were assessed by comparing TR-EPR spectra recorded on melem, oligomer, and CN samples, all in powder form. The corresponding schematic structures, derived from XPS analysis, are shown in Figure 2a,c,e. Figure 2b,d,f reports the TR-EPR spectra of the three materials recorded at 50 K and extracted between 1.0–1.3 or 1.3–1.5 μs after the 355 nm laser flash (Supporting Information section 4). The TR-EPR spectrum of melem (Figure 2b) displays a polarization pattern *eaeaea* (where *e* stands for emission and *a* for an enhanced absorption signal). Spectral simulations provide a quantitative determination of the ZFS parameters (*D* and *E*) and the triplet sublevel populations, Table 2. Since the sign of *D* cannot be experimentally determined, in all simulations it was assumed *D* as positive in analogy to literature data on planar aromatic systems such as porphyrins, which display an oblate symmetry. *E* was also assumed as positive, yielding an energy order for the triplet sublevels in zero-field as y > x > z. For melem the magnitude of *D* ≈ 2000 MHz falls in the range observed for molecular species, while a *E*/*D* ratio of ≈ 0.1 indicates a moderate rhombicity. Due to the absence of heavy atoms in the structure, the spin–spin interaction is assumed to be the sole contributor to the ZFS tensor. Therefore, analysis of the *D* parameter under the point-dipole approximation (*D* ∝ r^–3^) yields an interspin distance *r* ∼ 0.29 nm, which is smaller than the edge-to-edge dimension of the heptazine (∼0.68 nm). This indicates that the extent of spin density delocalization involves only part of the molecule, in analogy to what is found for triplet states in common organic molecules (e.g., porphyrins).^27^

**Figure 2:**
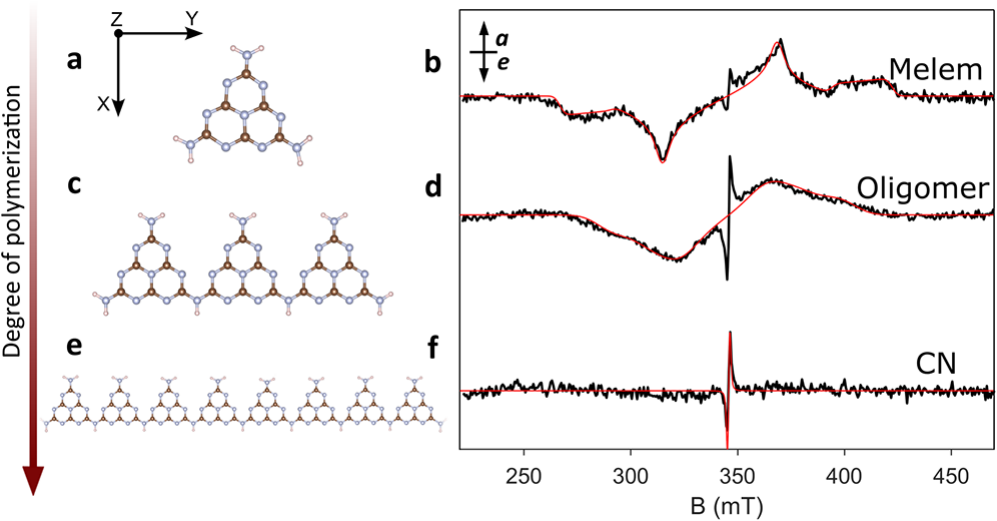
Structural representations of the melem (a), the oligomer (c), and CN (e), color code: blue, nitrogen; brown, carbon; white, hydrogen. The orientation of the ZFS tensor as assumed with respect to the molecular structure of melem is shown next to the molecular structure. TR-EPR spectra (black lines) and simulations (red lines) for melem (b), oligomers (d) and CN (f). The arrow on the left pictorially indicates the progressively higher temperatures employed in the synthesis of the three samples. All the TR-EPR measurements were performed at 50 K under irradiation at 355 nm. *a* = enhanced absorption, *e* = emission.

**Table 2:** Simulation Parameters Extracted from the TR-EPR Spectra of Melem, Oligomers and Polymeric CN Reported in Figure 2

***Sample***	***D*** **(MHz)**	***E*** **(MHz)**	***P***_***X***_ ***:P***_***Y***_ ***:P***_***z***_	***Linewidth (MHz)***
**melem**	2210 ± 20	225 ± 10	0.7:0.3:0	128
**oligomer**	1660 ± 20	180 ± 10	0.7:0.3:0	560
**CN**	30 ± 2	3 ± 2	0.63:0.38:0	25

The TR-EPR spectrum of the oligomers displays the same polarization pattern (*eaeaea*) but is characterized by a ZFS parameter *D* of ∼1600 MHz, which corresponds to a 25% decrement with respect to melem (Table 2 and Figure 2d). The characteristic turning points of a TR-EPR spectrum of a triplet exciton are less visible because of the large linewidth, which in the simulation was modeled as Gaussian. This is referred to as strain effects, which in solids reflect local structural distortions and are analogous to the conformational flexibility responsible for the inhomogeneous line broadening observed for molecular species in (frozen) solution.^28^

The alternative hypothesis, i.e. that the large linewidth is due to the simultaneous presence of two or more triplet excitons localized on oligomers of different length, can be ruled out since this would result in a series of well-defined spectra, each contributing to the final envelope with its own set of turning points. The spectrum of CN is consistent with those reported in the literature^23^ and is characterized by a significantly smaller *D* of about 30 MHz. Also in the case of CN, the polarization pattern is of the type *eaeaea*. In all cases, the polarization pattern and the ZFS parameter remain consistent up to room temperature, supporting the relevance of the TR-EPR experiments recorded at 50 K also to working conditions (Figure S7).

For all spectra, the spin polarization is characteristic of ISC mediated by vibronic coupling and corresponds to a preferential population of the two in-plane triplet spin sublevels (*p*_*x*_, *p*_*y*_) along the *x* and *y* ZFS axes. The axis of the largest dipolar interaction lies perpendicular to the melem plane, and it is inactive in terms of selective ISC population (*p*_*z*_). The spin polarization does not change significantly with increasing size, as already observed for other organic polymers (oligo­(*p*-phenyleneethynylene)­s).^29^

The narrow central spectral feature – which is attributed to the triplet exciton in polymeric CN^23^ – is also visible in the TR-EPR spectra of melem and of the oligomer (Figure 2). This is not unexpected, considering that both melem and oligomer contain also a fraction of polymerized material (see Figure 1d and Supporting Information section 3). In order to validate the assignment of this spectral feature to the polymeric phase, a comparison between the TR-EPR spectra of powder melem sample and melem dissolved in a DMSO/toluene solution is presented in Figure 3. Polymeric CN is known to be insoluble in DMSO as opposed to melem.^9^ The spectrum measured on the solution shows no trace of the narrow spectral feature, confirming the assignment to CN and demonstrating the possibility of detecting and differentiating different species through TR-EPR.

**Figure 3:**
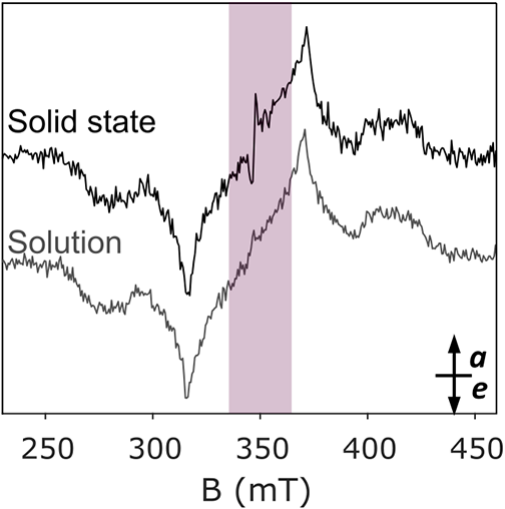
TR-EPR spectra recorded on melem powder (black) and on melem solution (50 mM of melem in a solvent mixture of DMSO:toluene 7:3 v:v) (gray). The spectra were recorded at 50 K under irradiation at 355 nm wavelength. *a* = enhanced absorption, *e* = emission.

By examination of Table 2, the following considerations emerge. First, the ZFS parameter *D* decreases as the number of units in the materials increases. Since *D* – in the point dipole approximation – is inversely proportional to the average distance between unpaired electrons, this indicates that CN has the greatest exciton delocalization among the materials considered. Second, the significant decrease in *D* from the oligomer (about 3 units) to CN (about 8 units) suggests a significant change in the electronic structure of the paramagnetic state over a size range of roughly 2 to 5 nm. Third, although *D* varies substantially across the series, the relative populations of triplet sublevels remain constant, implying that the molecular geometries are similar and that the mechanism of triplet exciton formation is conserved.

The properties of polymerized conjugated systems, such as CN, are strongly dependent on the intrinsic properties of the monomer, the size of the oligomers, and their morphology. Therefore, linking the photophysical properties to the number of units constituting the material is relevant to understand the charge generation and transport properties under light excitation and eventually the photochemistry displayed by the materials.^30^

The properties of the triplet excitons as observed by TR-EPR can be correlated with the optical properties of singlet excitons, as both depend on the electronic properties of the material and on the degree of delocalization. Melem and the oligomer are molecular species, and their photophysics is governed by the HOMO–LUMO gap. It is expected that the larger the number of melem units, the smaller the HOMO–LUMO gap. This can be estimated through photoluminescence, which is anticipated to undergo a redshift as a function of the number of units. CN is, on the other hand, a semiconductor better described by the gap between the valence and conduction bands, i.e. the bandgap (*E*_*g*_). Because of the low dielectric constant of CN (*ε*_*r*_ = 4–6),^31^ photogenerated electron and hole pairs are bonded by the Coulomb interaction. This attraction results in the creation of an exciton band below the threshold of the conduction band, from which the optical bandgap (*E*_*opt*_) is defined as the energy difference between the bandgap and the exciton binding energy (*E*_*b*_), according to *E*_*opt*_ *= E*_*g*_ *– E*_*b*_. In CN photoluminescence originates from the radiative decay of excitonic states, rather than band-edge states, due to strong excitonic effects.^32^

For conjugated polymers, it has been empirically shown that the fit of the HOMO–LUMO gap against the reciprocal of the number of monomer units (1/n) affords a linear relation,^33,34^ as expected from the simple particle-in-a-box model. The same trend has also been reported for the ZFS parameter *D* for the triplet exciton of simple conjugated systems such as thiophene oligomers^35^ and carotenoids,^36^ but not for more complex porphyrin oligomers.^27,37,38^

From the room-temperature photoluminescence spectra shown in Figure 1b, the HOMO–LUMO gap energies for melem and the oligomer, as well as the optical gap (E_opt_) of CN, were estimated from the emission maxima. Figure 4a presents the variation of the ZFS parameter *D* and the optical gap as a function of the inverse number of melem units (1/n). While neither parameter follows a linear trend with 1/n, a clear correlation emerges between the two, as shown in Figure 4b: larger optical gaps are associated with higher ZFS *D* values, consistent with a more localized triplet exciton. Although the number of data points is limited, the observed trend is suggestive and hints at a relationship between exciton localization and the degree of polymerization — highlighting the potential of this approach despite the synthetic challenges in accessing the full oligomeric series.

**Figure 4:**
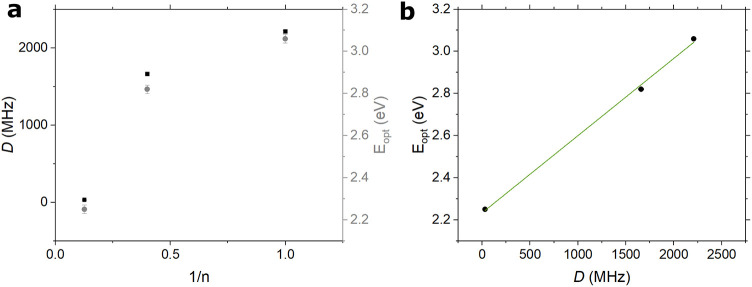
**a** – Dependence of the ZFS parameter *D* (black dots) and of the optical bandgap (gray dots) as a function of the number of monomeric units constituting the melem, oligomer, and CN materials. Whiskers indicate the estimated errors. **b** – Plot showing the linear correlation between the ZFS parameter *D* and the optical bandgap according to the equation E_opt_ = m*D* + q, where m = (3.666 × 10^–4^ ± 1.745 × 10^–5^) eV/MHz and q = (2.233 ± 0.028) eV, *R* ^2^ = 0.9977.

To summarize, in this work the properties of triplet excitons in CN-related materials were systematically investigated across a series of structurally defined systems with increasing dimensionality. By arresting the thermal condensation reaction at selected stages, we isolated and studied the monomer (melem), oligomers (estimated ∼3 monomeric units), and fully polymerized CN (estimated ∼8 monomeric units), enabling a stepwise analysis from molecular to extended macromolecular architectures.

A multitechnique approach — combining time-resolved electron paramagnetic resonance (TR-EPR), diffuse reflectance UV–vis, photoluminescence spectroscopy, ATR-IR, and X-ray photoelectron spectroscopy (XPS) — allowed comprehensive characterization of the structural and electronic properties of the materials. TR-EPR spectra provided direct experimental evidence for the formation of triplet excitons in all materials, with spectroscopic features unique to each level of polymerization.

The ZFS parameter *D*, which serves as a proxy for the spatial extent of the triplet exciton, decreased systematically with an increasing macromolecular size, indicating progressive delocalization of the exciton across the lattice. This trend correlates strongly with the optical gap, estimated from photoluminescence spectra, showing consistent behavior across both magnetic and optical measurements. Together, these results establish a clear link between polymerization degree, electronic structure, and exciton delocalization. Importantly, this study demonstrates that the triplet exciton observed in fully polymerized CN is an intrinsic feature of the polymer,^23^ not attributable to unreacted monomers or smaller condensates.

Although the role of triplet excitons is well established in organic electronics — for example, in triplet–triplet annihilation^39–42^ and singlet fission^43,44^ processes — their impact on the photophysics and photochemistry of CN materials remains largely unexplored. However, singlet–triplet inversion, predicted and observed in heptazine, melem, and CN, clearly points to the central role of triplet excitons in governing their excited-state dynamics.^16,23,45^ In this context, TR-EPR spectroscopy, with its unique selectivity to paramagnetic species and its time resolution on the scale of exciton diffusion and surface reactivity, proves to be a powerful and essential tool for bridging the current knowledge gap.

## Supplementary Material



## Notes

Description of the experimental procedures: sample preparation and characterization methods. Additional photoluminescence emission spectra and Rietveld refinement on the diffractogram of melem (Figure S1). Direct comparison between experimental and deposited XRD diffractograms (Figure S2). Time-correlated single photon counting time traces (Figure S3 and Table S1). Analysis of C1*s* XPS spectra as a function of polymerization (Figure S4) and semiquantitative analysis of the XPS spectra for melem, oligomer, and CN (Table S2). TR-EPR transients (Figure S5). TR-EPR simulation of CN (Figure S6). Room-temperature TR-EPR for melem, oligomer and CN (Figure S7). (PDF)

## †

Université Claude Bernard Lyon 1, CNRS, ENS Lyon, CRMN UMR 5082, 69100 Villeurbanne, France
