# Supplementary material for: Triplet Excitons in Carbon Nitride Materials: From Melem Monomers to Extended Polymers
Source: J Phys Chem Lett. 2025 Oct 12;16(42):10899–905. doi: 10.1021/acs.jpclett.5c02668 (PMC12557356; doi:10.1021/acs.jpclett.5c02668)
Supplement: Supplementary file 1 [file jz5c02668_si_001.pdf]

Supporting Information

Triplet Excitons in Carbon Nitride Materials: from Melem Monomers to Extended Polymers

Arianna Actis[a,‡], Niccolò Ulivieri[b,c], Lorenzo Poggini[b,c], Mario Chiesa[a], Enrico Salvadori[a,*]

[a]Dipartimento di Chimica, Università di Torino, Via P. Giuria 7, 10125 Torino (TO), Italy

[b]CNR, ISTITUTO DI CHIMICA DEI COMPOSTI ORGANOMETALLICI, Via Madonna del Piano 10, 50019 Sesto Fiorentino (FI), Italy

[c] Department of Chemistry 'Ugo Schiff' DICUS, and INSTM Research Unit, University of Florence, Via Della Lastruccia 3-13, 50019 Sesto Fiorentino, Italy

[‡]Present address: Université Claude Bernard Lyon 1, CNRS, ENS Lyon, CRMN UMR 5082, 69100 Villeurbanne, France

* Corresponding author: enrico.salvadori@unito.it

1. Materials and Methods

1.1 Sample preparation.

All reagents were purchased by Sigma Aldrich and used without further purification.

The $-NH_2$ terminated heptazine (melem) was synthesised by placing 2 g of melamine in a quartz vessel covered with a lid and heating it in a muffle furnace at 400°C for 5 hours with a 1°C/min temperature ramp with a procedure derived from[1]. The white product was then collected and milled. The oligomers were synthesised placing 2 g of melamine in a crucible covered with a lid in a muffle furnace and heating it for 12 h at 450°C with a 5°C/min temperature ramp following the procedure reported by Lau et al.[2] The white product was collected and milled. Polymeric CN material discussed in this work is the amorphous CN morphology (am-CN), synthesised from graphitic CN followed by a further thermal treatment, as discussed in refs[3,4]. Briefly, graphitic CN was obtained from 10 g of melamine heated in a muffle furnace at 550 °C in air for 300 minutes with a temperature ramp of 5 °C/min. The amorphous CN was then obtained with a post-synthetic treatment by heating 1 g of graphitic CN in a tubular furnace at 620 °C for 6 hours with a temperature ramp of 2 °C/min under continuous Ar flow. The final product was milled in order to obtain a uniform powder.

1.2 Sample Characterisation.

X-ray photoelectron spectroscopy. To perform XPS analysis, all the powdery samples were stuck by friction on a scratched and clean copper substrate and put under vacuum into an UHV XPS chamber (10^{-9} mbar). XPS data were acquired using a non-monochromatic X-ray source (VSW-TA10 Al Kα radiation, λ = 1486.6 eV), working at 120 W power (12 kV and 10 mA) and mounted at an angle of 54.44° with respect to the analyser (VSW-HA100 hemispherical analyser equipped with a 16-channel detector). The pass energy value was set to 44 eV in FAT (Fixed Analyser Transmission) mode. XPS spectra were calibrated with respect to the Cu $2p_{3/2}$ peak (932.61 eV) as an internal reference for copper (0)[5]. Linear and Shirley backgrounds were used as backgrounds and the spectra deconvolution was done using a combination of Gaussian and Lorentzian functions and the fitting of the spectra were done with CasaXPS software[6].

Powder XRD. Powder X-ray diffraction patterns were recorded with a PANalytical PW3040/60 X'Pert PRP MPD using a copper Kα radiation source of 0.154056 nm in a Bragg-Brentano geometry. The reflections were scanned continuously with 5° < 2θ< 80° and a resolution of 0.017°. X'Pert High-Score (Malvern Panalytical Ltd, Malvern, UK) and Materials Analysis Using Diffraction (MAUD) softwares were used to analyse the patterns.

The average size of the crystallites was estimated from the XRD pattern through the Scherrer equation:

$$d = \frac{K \cdot \lambda}{\beta \cdot cos\theta}$$

where d is the mean size of the ordered domains (crystallite); K is a dimensionless shape factor (taken to be equal to 0.9); λ is the working X-ray wavelength factor (0.154056 nm); β is the peak broadening (full width half maximum, FWHM), corrected for the instrumental broadening, expressed in radians; and θ is the Bragg angle. To estimate the average crystallite size, the (002) reflection at 2θ = 27.5° was used and the FWHM was measured to be 1.393°.

ATR-IR. Attenuated total reflection infrared spectroscopy (ATR-IR) spectra were acquired on Perkin-Elmer Spectrum Two FT-IR spectrometer with the ATR accessory with a diamond cell and a MIR source (8000-30 cm^{-1}). ATR were obtained recording 16 scans between 800 and 4000 cm^{-1} at a resolution of 4 cm^{-1}.

Diffuse Reflectance UV-VIS. The UV-VIS diffuse reflectance spectra were recorded using a Varian Cary 5000 spectrophotometer (Agilent, CA, USA) equipped with an integration sphere for diffuse reflectance (DR) studies, using the Carywin-UV/scan as software (Agilent, CA, USA). A Teflon sample with 100% reflectance was used as reference. All the measurements were conducted on the samples in powder form at room temperature.

Photoluminescence spectroscopy. Steady state emission spectra were collected on a Horiba Jobin Yvon Fluorolog 3 equipped with a 450-W Xenon lamp using the FluorEssenceTM software. Spectra were recorded at different excitation wavelengths. Spectral resolution is 1 nm, while both excitation and emission slits were set at either 3 or 4 nm. All the measurements were conducted on the samples in powder form at room temperature.

Transient photoluminescence was measured with a Time-Correlated Single Photon Counting (TCSPC) setup (Horiba Jobin Yvon Fluorolog3). Pulsed 370 nm excitation (100 kHz repetition rate) was generated by a laser diode (SpectraLED) and fluorescence was detected at 540 nm, 470 nm and 420 nm for CN, oligomer and melem respectively (TBX-4 detector). Data were collected into 2048 channels, to 10,000 counts in the peak channel. Emission decay data were analyzed using the software package DAS6 (TCSPC Decay Analysis Software). Powder samples were measured at room temperature in air atmosphere.

Time-Resolved EPR spectroscopy. X-band (microwave frequency 9.76 GHz) TR-EPR experiments were performed at 50 K on a Bruker ELEXYS 580 EPR spectrometer equipped with a Bruker ER 4118X-MD5 resonator, a cryo-free helium cryostat from Cryogenics. The magnetic field was measured by means of a Bruker ER035 M NMR gaussmeter. Optical excitation of the sample was performed in the EPR cavity by means of a UV-Vis tunable OPO laser AURORA II (Litron), with a repetition rate of 21 Hz, pulse length 7 ns and approximate energy per pulse 2 mJ. The TR-EPR measurements were performed in direct detection mode using the Bruker SpecJetTM transient recorder. The microwave power was set to 6.29 mW. For each field position, a time trace with 1024 points and 100 or 200 transient averages was recorded using a time base of 4 ns. All the measurements were conducted on the samples in powder form at 50 K.

2. **Structural characterization.**

Figure S1 a,b,c reports additional photoluminescence data for melem, oligomer and CN. The melem sample and the oligomers were subjected to a complementary structural characterisation which was then compared with literature data. Figure S1d shows the powder X-ray diffractogram obtained on melem and analysed through the Rietveld refinement to estimate the ratio between the melem phase and the polymeric CN. The values obtained amount to ca. 74 % of melem and 26 % of CN. Similar analysis could not be performed on the sample constituted by the oligomers, since there is no deposited diffractogram of any oligomer. However, visual inspection in the diffractogram shows clear presence of broad diffraction peaks at $2\theta \sim 13°$ and $2\theta \sim 27°$, typical of polymeric CN, Figure S2. Besides, the diffractogram also displays some sharper reflections analogous to those reported by Lotsch et al.[2] and attributed to smaller condensates.

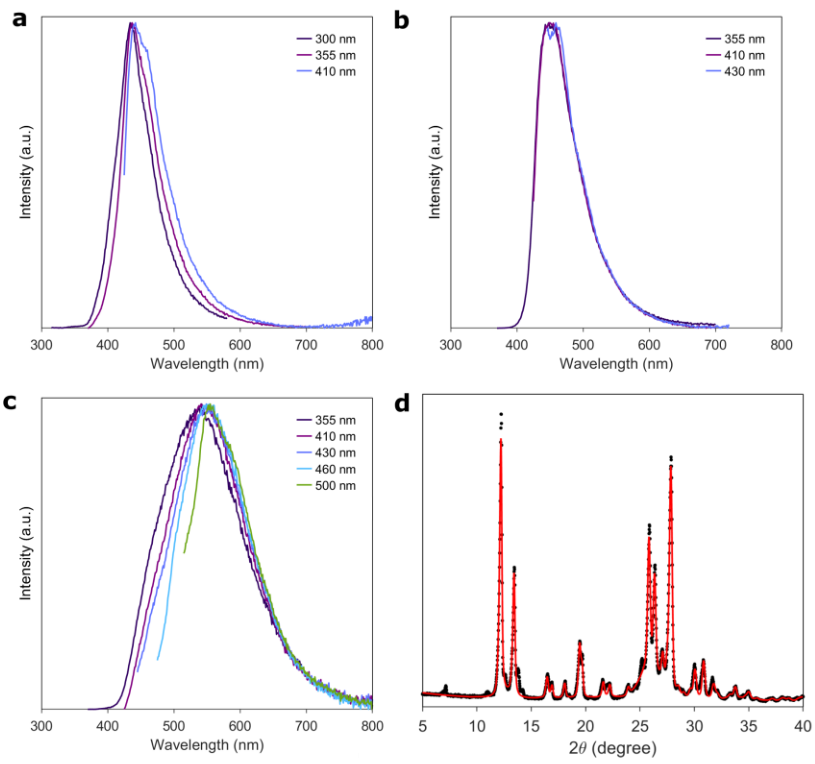

Figure S1. Photoluminescence emission spectra measured on a) melem, b) oligomer and c) CN powders. Excitation wavelengths are indicated in each panel. d) Rietveld refinement on the XRD diffractogram of melem sample.

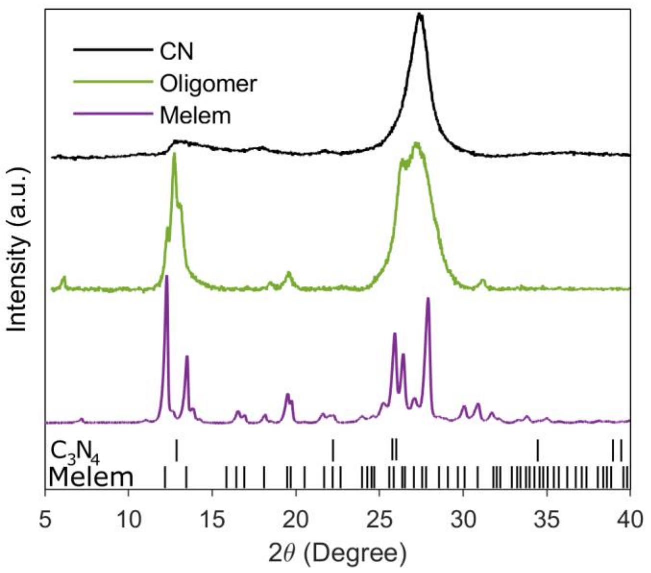

Figure S2. Comparison between the experimental XRD diffractograms of melem, oligomer and CN together with the reference peak positions for C_3N_4 (CCDC identifier 1703405[7]) and melem (COD identifier 4114092[1]).

Figure S3 reports the TCSPC traces for melem, oligomer and CN. The fluorescence lifetime values extracted from biexponential fitting (Table S1) are in line with previous literature reports[8,9].

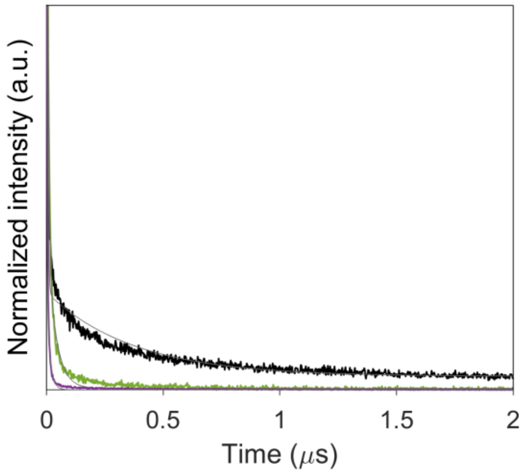

Figure S3. Experimental TCSPC traces for melem (purple line), oligomer (green line) and CN (black line). The corresponding biexponential fittings are displayed as grey lines. The fitting parameters are reported in Table S3.

Table S1. Biexponential fitting parameters of the TCSPC traces for melem, oligomer and CN

	Melem		Oligomer		CN	
**Component**	Amplitude	Time (ns)	Amplitude	Time (ns)	Amplitude	Time (ns)
Fast	0.891±0.005	1.87±0.02	0.81±0.01	5.26±0.12	0.872±0.013	3.27 ±0.10
Slow	0.109±0.005	13.3±0.5	0.17±0.01	36.2±1.7	0.088 ±0.002	371.8 ±17.3

3. C1*s* XPS spectra

Table S2 reports the semiquantitative analysis of the XPS spectra for melem, oligomer, CN.

The C1*s* region of the XPS spectra provides limited insight regarding the actual structure of the samples due to the similarities of the XPS peak positions across the samples. However, for completeness we report here a description of the relevant features and the corresponding deconvolutions (Figure S4).

The C1s XPS spectrum of melem exhibits a dominant peak at 289.35 eV, which is consistent with the presence of aromatic carbon within the tris-triazine structure. A secondary peak centered at 287.10 eV is likely due to surface contaminants, as also reported in the literature[10,11]. Additionally, the signal observed at 285.51 eV is commonly associated with adventitious carbon, typically arising from environmental exposure.

The C1*s* spectra for the oligomer sample closely resembles that of melem. The most intense peak, centered at 289.01 eV, is consistent with the presence of aromatic carbon atoms within the structure. The intermediate signal at 287.00 eV can be attributed to both oxygen incorporation into the carbon

nitride framework and to surface contaminants. Additionally, the signal at 285.46 eV corresponds to adventitious carbon, commonly observed due to environmental exposure.

The C1*s* spectrum of CN reveals three distinct components, consistent with those observed in both melem and the oligomer. These include a peak at 285.31 eV associated with aromatic carbon, a signal at 287.21 eV attributed to contaminants and/or oxygen incorporation, and a contribution from adventitious carbon, inadvertently introduced during sample handling or exposure.

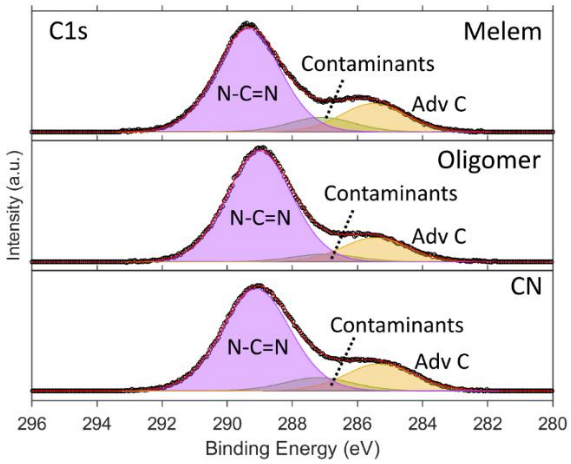

Figure S4. C1*s* XPS spectra as a function of polymerization.

Table S2: Semiquantitative analysis of the XPS spectra for melem, oligomer, CN.

Sample	C1*s* B.E. (eV)/[Area (%)]	N1*s* B.E. (eV)/ [Area (%)]
**melem**	C adv 285.51 / [19.5]	N py 399.70 / [56.4]
	contaminants 287.10 / [10.0]	NH_2 400.50 / [28.2]
	N-C=N 289.35 / [70.5]	N-$(C)_3$ 402.10 / [9.4]
		charging 406.36 / [6.0]
**oligomer**	C adv 285.46 [17.0]	N py 399.52 [44.2]
	contaminants 287.00 [5.4]	NH_2 400.32 [24.8]
	N=C-N 289.01 [77.6]	NH 401.12 [8.1]
		N-$(C)_3$ 402.02 [11.8]
		charging 405.30 [7.4]
		NH_3 397.87 [3.8]
**CN**	C adv 285.31 [19.0]	N py 399.54 [57.6]
	contaminants 287.21 [9.3]	NH_2 400.34 [13.4]
	N=C-N 289.11 [71.7]	NH 401.14 [9.2]
		N-$(C)_3$ 402.04 [10.6]
		charging 405.62 [9.2]

4. TR-EPR Spectra Analysis.

The background signal was removed by a linear 2D baseline correction using the signal acquired before the laser pulse for the time-dimension and the high- and low-field off-resonant transients for the field dimension. To increase the signal-to-noise ratio, the corrected spectra were integrated over the time window 1.0-1.3 μs (CN) and 1.3-1.5 μs (oligomer and melem) after the laser pulse, as within this range no significant change in the signal shape or intensity was observed, Figure S5.

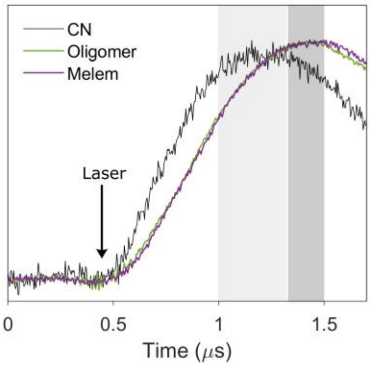

Figure S5. Normalized TR-EPR transients of melem, oligomer and CN excited at 355 nm (50 K). The shaded areas show the integration windows used for CN (1.0-1.3 μs, 348 mT) and melem and oligomer (1.3-1.5 μs, 371 mT). No significant evolution occurs within either integration windows.

The spectra were simulated with EasySpin[12]. The TR-EPR spectra were modelled with a spin Hamiltonian describing a triplet spin state in an external applied magnetic field B_0:

$$\hat{H} = \mu_B g \hat{S} \cdot B_0 + D\left(\hat{S}_z^2 - \frac{1}{3}S(S+1)\right) + E\left(\hat{S}_x^2 - \hat{S}_y^2\right)$$

Where the first term is the electron Zeeman interaction, the second and third terms describe the zero-field splitting interaction. The *g* factor is assumed isotropic and equal to the free-electron *g* value for melem and oligomer (g_e = 2.0023) and 2.0032 for CN[4]. *D* and *E* parametrise the zero-field splitting interaction as follows:

$$D = \frac{3}{4}\frac{\mu_0}{4\pi h}(g_{iso}\mu_B)^2\langle\frac{r^2-3z^2}{r^5}\rangle = \frac{3}{4}\frac{\mu_0}{4\pi h}(g_{iso}\mu_B)^2\langle\frac{1-3cos^2\theta}{r^3}\rangle \quad \text{Eq.S1}$$

$$E = \frac{3}{4}\frac{\mu_0}{4\pi h}(g_{iso}\mu_B)^2\langle\frac{y^2-x^2}{r^5}\rangle \quad \text{Eq.S2}$$

where *r* is the distance between the electron spins, θ is the angle between the spin-spin vector *r* and the dipolar *Z* axis, g_{iso} is the isotropic *g* value for the system and *x, y, z* are the principal axes of the ZFS interaction. The angular brackets indicate the expectation value taken over the triplet wavefunction. It follows that *D* can be related to the average inter-spin distance, whereas the parameter *E* contains information about the rhombicity of the ZFS tensor. *D* represents the distortion along the axial direction of the ZFS tensor, and its sign depends on the (1-3cos^{2}θ) term, indicating whether the spin distribution is “disk-like” (oblate, θ = 90°, *D* > 0) or “rod-like” (prolate, θ = 0°, *D* < 0). In the former case the *Z* axis of maximum dipolar coupling is perpendicular to the plane of the spin distribution, whereas in the latter the axis of maximum dipolar coupling (*Z*) is parallel to the long axis of the spin distribution. Given the flat structure of heptazine and CN, it is expected that the spin distribution will be “disk-like” therefore the ZFS parameter *D* has been considered as > 0 (and therefore θ = 90°). Since both D and E are assumed as positive in the simulations, the energy

ordering of the triplet sublevels is y> x> z. Figure S6 shows the simulation of the TR-EPR spectrum of CN.

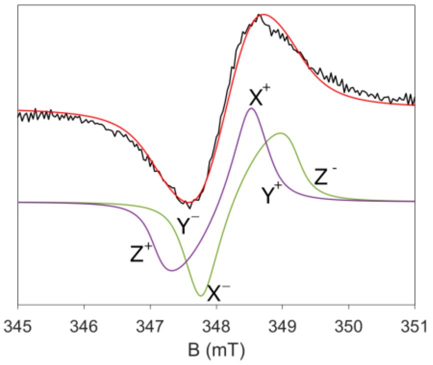

Figure S6. Experimental TR-EPR spectrum of CN recorded at 50 K (black line) together with the corresponding simulation (red line). The positions of the six canonical transitions and the contribution of each transition are also reported: $m_S = -1 \leftrightarrow m_S = 0$ (green line) and $m_S = 0 \leftrightarrow m_S = +1$ (purple line). Simulation parameters: D = +30 MHz, E = +3 MHz, Px:Py:Pz = 0.63:0.38:0.

The optimal temperature for TR-EPR measurements is mainly determined by the spin lattice relaxation, which controls the time-evolution of the triplet state sublevel populations. Ideally, this should be slow so that a high spin polarization is maintained throughout the experiment. Figure S7 shows that the polarization pattern and the ZFS parameter remain unchanged up to room temperature supporting the relevance of the TR-EPR experiments recorded at 50 K also to working conditions.

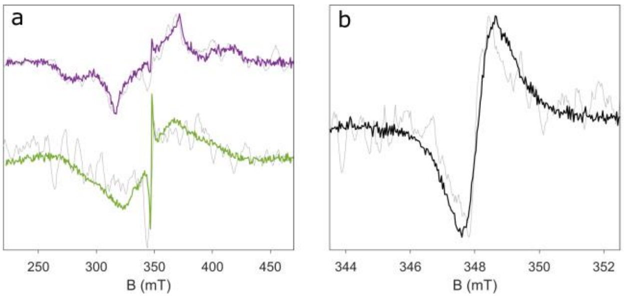

Figure S7. a) TR-EPR spectra recorded at room temperature (grey) and at 50 K (purple and green) on melem and oligomer samples respectively; b) TR-EPR spectra recorded at room temperature (grey) and at 50 K (black) on CN. Room temperature spectra were smoothed with a 2nd order Savitzky-Golay filter (5-point window).

---

[1] Jurgens B., Irran E., Senker J., Kroll P., Muller H., Schnick W., Melem (2, 5, 8-triamino-tri-s-triazine), an important intermediate during condensation of melamine rings to graphitic carbon nitride: synthesis, structure determination by x-ray powder diffractometry, solid-state nmr, and theoretical studies, *J. Am. Chem. Soc.*, **2003**, *125*, 34, 10288–10300

[2] Lau V. W.-h., Mesch M. B., Duppel V., Blum V., Senker J., Lotsch B. V., Low molecular- weight carbon nitrides for solar hydrogen evolution. *J. Am. Chem. Soc.*, **2015**, *137*, 3,1064–1072

[3] Filippini G., Longobardo F., Forster L., Criado A., Di Carmine G., Nasi L., D'Agostino C., Melchionna M., Fornasiero P., Prato M., Light-driven, heterogeneous organocatalysts for c–c bond formation toward valuable perfluoroalkylated intermediates. *Sci. Adv.,* **2020**, *6*, 46, eabc9923

[4] Actis A., Melchionna M., Filippini G., Fornasiero P., Prato M., Chiesa M., Salvadori E., Singlet-Triplet Energy Inversion in Carbon Nitride Photocatalysts, *Angew. Chem. Int. Ed.*, **2023**, e202313540

[5] Biesinger, M. C., Lau, L. W. M., Gerson, A. R., Smart, R. S. C. Resolving surface chemical states in XPS analysis of first row transition metals, oxides and hydroxides: Sc, Ti, V, Cu and Zn. *Appl. Surf. Sci.,* **2010**, *257*, 887–898

[6] Fairley N., Fernandez V., Richard-Plouet M., Guillot-Deudon C., Walton J., Smith E., Flahaut D., Greiner M., Biesinger M., Tougaard S., Morgan D., Baltrusaitis J., Systematic and collaborative approach to problem solving using X-ray photoelectron spectroscopy. *Appl. Surf. Sci. Adv.* **2021**, *5*, 100112

[7] Fina F., Callear S. K., Carins G. M., Irvine J. T. S., Structural Investigation of Graphitic Carbon Nitride via XRD and Neutron Diffraction, *Chem. Mat.*, **2015**, *27*, 2612

[8] Godin, R., Wang Y., Zwijnenburg M.A., Tang J., Durrant J.R., Time-Resolved Spectroscopic Investigation of Charge Trapping in Carbon Nitride Photocatalysts for Hydrogen Generation, *J. Am. Chem. Soc*. **2017**, *139*, 14, 5216–5224

[9] Aizawa N., Pu YJ., Harabuchi Y, Nihonyanagi A., Ibuka R., Inuzuka H., Dhara B., Koyama Y., Nakayama K., Maeda S., Araoka F., Miyajima D., Delayed fluorescence from inverted singlet and triplet excited states. *Nature,* **2022**, *609*, 502–506

[10] Cometto, C., Ugolotti A., Grazietti E., Moretto A., Bottaro G., Armelao L., Di Valentin C., Calvillo L., Granozzi G., Copper single-atoms embedded in 2D graphitic carbon nitride for the CO2 reduction. *Npj 2D Mater. Appl.*, **2021**, *5*, 1–10

[11] Akaike, K., Aoyama, K., Dekubo, S., Onishi, A., Kanai, K. Characterizing Electronic Structure near the Energy Gap of Graphitic Carbon Nitride Based on Rational Interpretation of Chemical Analysis. *Chem. Mater.*, **2018**, *30*, 7, 2341–2352 2341–2352

[12] Stoll S., Schweiger A., EasySpin, a comprehensive software package for spectral simulation and analysis in EPR. J*. Magn. Res.*, **2006**, *178*, 1, 42–55
